# Letter to the Editor: Enhancing COVID-19 vaccination coverage using financial incentives: arguments to help health providers counterbalance erroneous claims

**DOI:** 10.4178/epih.e2021087

**Published:** 2021-10-22

**Authors:** Yong-jun Choi

**Affiliations:** Department of Social and Preventive Medicine, Hallym University College of Medicine, Chuncheon, Korea

Dear Editor,

Dotlic et al. [1] described the cash offer approach taken by the Serbian government to improve the coronavirus disease 2019 (COVID-19) vaccination rate. They focused on refuting ethical criticisms of this approach. I would like to comment on some points.

First, the argument that a cash offer for vaccination is not different from the COVID-19-related financial hardship relief program needs theoretical support. Behavioral scientists may criticize this argument based on the concept of ‘mental accounting,’ according to which people tend to treat money differently depending upon its origin and other factors [2]. This concept suggests that rewards for vaccination and financial support for economic difficulty may be perceived as different in nature.

Their second argument is an example of the straw man fallacy. Few experts criticize cash offers for vaccination by referring to the problem of payment for participation in a clinical trial. In addition, the argument that financial rewards are not a form of coercion may be countered by the rebuttal that, in practice, they work as such for low-income people [3, Jecker’s second comment]. During the COVID-19 pandemic, coercive measures such as lockdowns and social distancing have been imposed on people in many countries, worsening their economic hardship. This may lead the unvaccinated to get immunized due to the cash offer despite doubts about vaccination or against their will. How could this approach be ethically justified if people change their minds upon learning about the cash offer?

Lastly, public health experts and government agencies have publicized the benefits of vaccination, but those who are not vaccinated may not be fully convinced. If this is the case, it is first necessary to identify the reasons for this phenomenon. In other words, one needs to figure out why people are reluctant to get vaccinated in order to devise an appropriate solution. We should never refrain from assessing the appropriateness of a policy measure only because the situation is severe. Limited resources must be used efficiently, and the COVID-19 pandemic may not be the last crisis of this sort.

The Serbian government’s attempt is interesting. However, its effectiveness should be empirically evaluated, and its ethical justification must be rigorously proven. A short essay is insufficient for these purposes.
